# Subretinal electronic chips allow blind patients to read letters and combine them to words

**DOI:** 10.1098/rspb.2010.1747

**Published:** 2010-11-03

**Authors:** Eberhart Zrenner, Karl Ulrich Bartz-Schmidt, Heval Benav, Dorothea Besch, Anna Bruckmann, Veit-Peter Gabel, Florian Gekeler, Udo Greppmaier, Alex Harscher, Steffen Kibbel, Johannes Koch, Akos Kusnyerik, Tobias Peters, Katarina Stingl, Helmut Sachs, Alfred Stett, Peter Szurman, Barbara Wilhelm, Robert Wilke

**Affiliations:** 1 Centre for Ophthalmology, University of Tübingen, Schleichstr. 12, 72076 Tübingen, Germany; 2 Eye Clinic, University of Regensburg, Franz-Josef-Strauss-Allee 11, 93053 Regensburg, Germany; 3 Retina Implant AG, Gerhard-Kindler-Str. 8, 72770 Reutlingen, Germany; 4 Department of Ophthalmology, Semmelweis University, Tomo u. 25-29, 1083 Budapest, Hungary; 5 Steinbeis Transfer Centre Eyetrial at the Centre for Ophthalmology, Schleichstr. 12-16, 72076 Tübingen, Germany; 6 Klinikum Friedrichstadt, Friedrichstr. 41, 01067 Dresden, Germany; 7 NMI Natural and Medical Sciences Institute at the University of Tübingen, Markwiesenstr. 55, 72770 Reutlingen, Germany

**Keywords:** subretinal neuro-prosthetics, retinal implant, retinitis pigmentosa, blindness, artificial vision, bionic vision

## Abstract

A light-sensitive, externally powered microchip was surgically implanted subretinally near the macular region of volunteers blind from hereditary retinal dystrophy. The implant contains an array of 1500 active microphotodiodes (‘chip’), each with its own amplifier and local stimulation electrode. At the implant's tip, another array of 16 wire-connected electrodes allows light-independent direct stimulation and testing of the neuron–electrode interface. Visual scenes are projected naturally through the eye's lens onto the chip under the transparent retina. The chip generates a corresponding pattern of 38 × 40 pixels, each releasing light-intensity-dependent electric stimulation pulses. Subsequently, three previously blind persons could locate bright objects on a dark table, two of whom could discern grating patterns. One of these patients was able to correctly describe and name objects like a fork or knife on a table, geometric patterns, different kinds of fruit and discern shades of grey with only 15 per cent contrast. Without a training period, the regained visual functions enabled him to localize and approach persons in a room freely and to read large letters as complete words after several years of blindness. These results demonstrate for the first time that subretinal micro-electrode arrays with 1500 photodiodes can create detailed meaningful visual perception in previously blind individuals.

## 1. Introduction

Retinitis pigmentosa (RP) and age-related macular degeneration are diseases that predominantly affect photoreceptors of the retina and cause progressive vision loss—leading eventually to blindness in over 15 million people worldwide [1]. Although blindness owing to photoreceptor degeneration presently remains incurable, inner retinal nerve cells may continue to function for many years despite neuronal remodelling [2]. While gene therapy and application of neuro-protective factors may help maintain vision in the early stages of degeneration, survival of the inner retina encouraged us [3] and others [4–11] to attempt a partial restoration of visual function using electric stimulation of the remaining retinal network.

Two fundamentally different approaches have been taken in this area: (i) implantation of electrode arrays which interface epiretinally with retinal ganglion cells that form the retinal output pathway [6–7,11–13], and (ii) implantation of microchips under the transparent retina to substitute the degenerated photoreceptors. The latter type of microchip senses light and generates stimulation signals simultaneously at many pixel locations, using microphotodiode arrays (MPDAs; [3,14]). While the first approach typically requires external image and data processing due to bypassing retinal image analysis, the second seeks to replace the function of degenerated photoreceptors directly by translating the light of the image falling onto the retina point by point into small currents that are proportional to the light stimulus. Ours is the only approach where the photodiode–amplifier–electrode set is contained within a single pixel of the MPDA such that each electrode provides an electrical stimulus to the remaining neurons nearby, thereby reflecting the visual signal that would normally be received via the corresponding, degenerated photoreceptor.

On the basis of *in vitro* measurements [15] and animal studies [16] our consortium developed a subretinal electronic implant that carefully accounts for biocompatibility [17], biostability, surgical feasibility by means of a transchoroidal surgical technique [18], safe threshold stimulation and dynamic range of stimulation and the limits of spatial resolution *in vitro* [19]. This report describes the results of a clinical pilot study, illustrating that subretinally implanted multi-electrode arrays restore sufficient visual function for object recognition and localization and for the performance of visual tasks essential in the daily lives of blind patients. The results of this pilot study provide strong evidence that the visual functions of patients blinded by a hereditary retinal dystrophy can, in principle, be restored to a degree sufficient for use in daily life.

## 2. The subretinal implant

**Figure 1.**
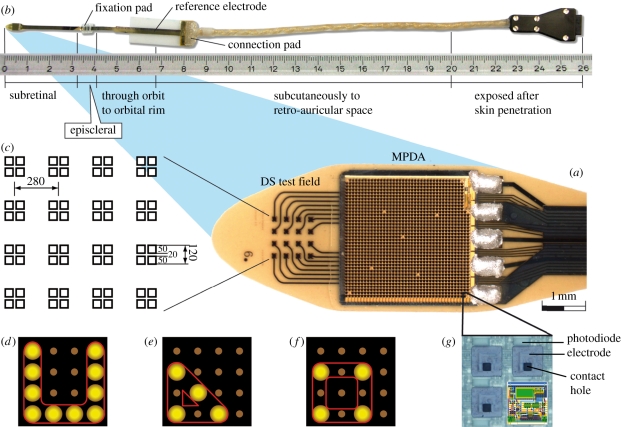
Subretinal implant. (*a*) The microphotodiode array (MPDA) is a light sensitive 3.0 × 3.1 mm CMOS-chip with 1500 pixel-generating elements on a 20 µm thick polyimide foil carrying an additional test field with 16 electrodes for direct electrical stimulation (DS test field). (*b*) The foil exits approximately 25 mm away from the tip at the equator of the eyeball and is attached to the sclera by means of a small fixation pad looping through the orbit to a subcutaneous silicone cable that connects via a plug behind the ear to a power control unit. (*c*) Magnification of the DS electrode array showing the 16 quadruple electrodes and their dimensions. (*d*) Pattern stimulation via DS array (e.g. ‘U’). (*e*,*f*) switching from a triangle to a square by shifting stimulation of a single electrode. (*g*) Magnification of four of the 1500 elements (‘pixels’), showing the rectangular photodiodes above each squared electrode and its contact hole that connects it to the amplifier circuit (overlaid sketch).

**Figure 2.**
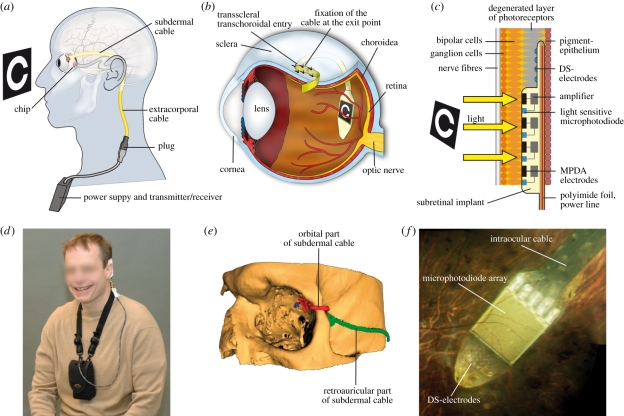
Implant position in the body. (*a*) The cable from the implanted chip in the eye leads under the temporal muscle to the exit behind the ear, and connects with a wirelessly operated power control unit. (*b*) Position of the implant under the transparent retina. (*c*) MPDA photodiodes, amplifiers and electrodes in relation to retinal neurons and pigment epithelium. (*d*) Patient with wireless control unit attached to a neckband. (*e*) Route of the polyimide foil (red) and cable (green) in the orbit in a three-dimensional reconstruction of CT scans. (*f*) Photograph of the subretinal implant's tip at the posterior eye pole through a patient's pupil.

As shown in figure 1*a*, the tip of the implant consists of an MPDA with 1500 individual light-sensitive elements and a test field for direct stimulation (DS) with 4 × 4 electrodes for electrical, light-independent stimulation. Both are positioned on a thin polyimide foil (figure 1*b*, far left). For details on the control unit that provides power and wireless control signals, see figure 2*a*,*d* and electronic supplementary material, chapter 1*c*.

### (a) The microphotodiode array

Each of the 1500 MPDA elements acts independently from its neighbours; four magnified elements (72 × 72 µm each) are shown in figure 1*g*. Each element includes a light-sensitive photodiode (15 × 30 µm) that controls a differential amplifier (circuit shown as a sketch) whose output stage is coupled to a titanium nitride (TiN) electrode (50 × 50 µm), connected to the amplifier via the contact hole (details see electronic supplementary material, chapter 1*b*). Essentially, an image is captured several times per second simultaneously by all photodiodes. Each element (‘pixel’) generates monophasic anodic voltage pulses at its electrode. Thus, pixelized repetitive stimulation is delivered simultaneously by all electrodes to adjacent groups of bipolar cells [15,19], the amount of current provided by each electrode being dependent on the brightness at each photodiode. Light levels ranging across approximately 2 log units are converted to charge pulses by each pixel with a sigmoidal relationship and the sensitivity can be shifted manually by several log units (see electronic supplementary material, chapter 1, figure S1). The chip is estimated to cover a visual angle of approximately 11° by 11° (1° approx. 288 µm on the retina). The distance between two MPDA electrodes corresponds to a visual angle of 15 min of arc.

### (b) The 4 by 4 test field for direct stimulation (DS test field)

The DS test field consists of 4 × 4 quadruple TiN electrodes (100 × 100 µm^2^, 280 µm apart laterally and 396 µm diagonally) for light-independent electrical stimulation (see figure 1*c*). The DS test field was added for assessment of the electrode-interface characteristics and to study current injections and efficacy of pulses with different shapes and polarities other than those provided by the MPDA. In a limited spatial testing range simple patterns can be created with the DS test field as well (figure 1*d*,*e* and *f*).

Threshold voltage to elicit a percept was assessed in an up-and-down staircase procedure. Typical charge transfer of a single electrode at threshold was between 20 and 60 nC per pulse (for details, see electronic supplementary material, chapter 1*a*). The maximum charge density at the electrodes in the DS field was 600 µC cm^−2^. These values were well within commonly accepted safety limits and have been proven safe even for continuous retinal stimulation *ex vivo* [20].

Impedance values of single electrodes were typically 300 kΩ (at 1 kHz sinusoidal AC). Although regular impedance measurements in the patients were not conclusive, analysis of all available data showed that charge thresholds, but not voltage thresholds decreased significantly during the first days after implantation. Thereafter, both charge and voltage thresholds showed a slight tendency towards increasing values over the remaining implantation period.

## 3. Patients

The patients (two males and one female, age 40, 44 and 38, respectively) were blind owing to hereditary retinal degeneration (patients 1 and 2: RP, patient 3: choroideraemia) but had good central vision previously. Disease onset was reported by patient 2 at age 16, by patients 1 and 3 at age 6. They had lost their reading ability at least 5 years before implantation. Bright light stimulation mediated some limited light perception without any recognition of shapes in all three patients. They reported neither general diseases nor regular medication (for details see electronic supplementary material, chapter 2*c*).

## 4. Methods

### (a) Surgical procedure

The implant, protected by a long steel tube, was advanced through a retroauricular incision to the lateral orbital rim and guided inside the orbit to the surface of the eyeball ([21]; figure 2*a*,*b*,*e*). The silicone cable (figure 2*a*) was implanted subperiostally beneath the temporal muscle. The polyimide foil was then protected by a silicone tube and guided from the lateral orbital rim, where it was fixed, to the equator of the eye. Subsequently pars plana vitrectomy was performed. A localized retinal detachment was created by saline injection in the upper temporal quadrant above the planned scleral and choroidal incision area. After preparation of a scleral flap, the implant was advanced *ab externo* transchoroidally along a guiding foil into the subretinal space until it reached the preoperatively defined position ([22]; see electronic supplementary material, chapter 2*d*). Although putting a chip directly under the fovea has not turned out to be a surgical problem we had abstained in initial patients from placing the chip under the macula, but asked to place the chip closer and closer to the foveola as the surgical learning curve improved. Silicone oil was then injected into the vitreous cavity to support retinal reattachment. No serious adverse events were noted during the course of the study. For post-operative observations and consideration on surgical safety see electronic supplementary material, chapter 2*f*).

### (b) Psychophysical tests

Beginning 7 to 9 days after surgery, tests with solely electrical stimuli were performed with the DS test field. Thereafter light evoked visual functions mediated by the MPDA-array were assessed using four psychophysical tests concerning light detection, basic temporal resolution, object localization and movement detection using the ‘basic light and motion test’ (BaLM [23]) described in electronic supplementary material, chapter 2*g*.

**Figure 3.**
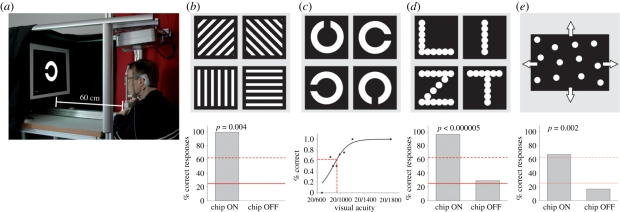
Recognition of projected targets (set up 1) (*a*) Set up for projecting targets on a screen. (*b*) Gratings of variable width, distance and luminance, presented individually in a ‘four-alternative forced choice’ mode (4AFC). (*c*) Landolt ‘C’ ring used in clinical tests of visual acuity. (*d*) Letters (8.5 cm high, 1.7 cm line width). (*e*) Random dot pattern moving in four different directions to assess spatio-temporal resolution. The inserts under each panel show the best results of patient 2 with the chip turned on and chip turned off. Solid line, chance rate; dashed line, psychometrically accepted recognition threshold; probability *p* as estimated from the binomial function.

**Figure 4.**
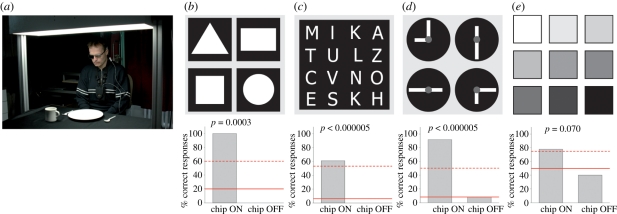
Recognition of objects (set up 2) (*a*) White items on a homogeneously illuminated black tablecloth. (*b*) Differentiation of four geometric objects with identical surface areas. (*c*) Differentiation of capital letters (height 5–8 cm). (*d*) Clock face for testing angle and size recognition. (*e*) Cards of different luminance presented in pairs to determine contrast vision. The respective inserts under each panel show the best results of patient 2 with the chip turned on and off. Solid line, chance rate; dashed line, psychometrically accepted recognition threshold; probability *p* as estimated from the binomial function (see electronic supplementary material, chapter 3*b*).

If passed successfully, three further steps followed: tests for recognition of stripe patterns (BAGA [24]), localization and recognition of objects common to daily life and visual acuity assessment (Landolt-C rings presented in an up-and-down staircase procedure to estimate the visual acuity in terms of maximum likelihood by means of FrACT test [25]). If these tasks were completed successfully, more challenging tasks were set (figures 3*a* and 4*a*). Except for some optional tasks (indicated) well-established two- or four-alternative forced-choice methods (2AFC and 4AFC, respectively) were employed in order to test for statistical significance of a patient's performance. All tests were performed separately in two conditions: with ‘Power ON’ and ‘Power OFF’ (‘baseline performance’).

Maximum screen luminance was approximately 3200 cd m^−2^ (for white light), neutral density filters (Schott NG filters 0.15–4 log U) served for attenuation (for details see electronic supplementary material, chapter 2).

## 5. Results

### (a) Electrical stimulation for pre-testing and learning via DS test field

Pulses of varying duration, polarity and shape were applied via the DS test field (16 electrodes, as shown in figure 1*c*–*f*) in a pre-testing routine. This procedure determined voltage thresholds for perception, accustomed the patients to electrically evoked visual impressions and tested retinal excitability and spatial resolution. An overview of the results including their statistical evaluation is given in a table presented in electronic supplementary material, chapter 3*a*.

All patients detected single-electrode single-pulse stimulation (0.5–6 ms pulses, typically 20–60 nC per electrode). Patients 1 and 2 consistently reported these stimuli as whitish round dot-like percepts, patient 3 reported percepts as elongated, short whitish/yellowish lines. Upon activation of four electrodes with a single pulse, all three patients correctly distinguished vertical lines from horizontal lines within seconds and spontaneously reported them as straight. Patients 1 and 2 distinguished multiple single dots upon simultaneous activation of several electrodes in a diagonal row and reported dark areas separating the dots. Patient 3 saw diagonal lines formed by four electrodes, but not the dark areas between the dots.

Simple patterns were also presented with the DS-array by pulsing electrodes sequentially (figure 1*d*); each electrode was switched on for 3–6 ms at intervals of 208 ms. Patients 1 and 2 correctly reproduced these patterns after the first single presentation; patient 3 failed to do so. Upon presentation of a four-alternative, forced-choice (4AFC) paradigm, patients 1 and 2 reliably differentiated four different positions of the opening of the letter ‘U’ (73% and 88% correct responses, respectively, see electronic supplementary material, chapter 5, movie 1). Furthermore, patient 1 correctly distinguished ‘U’ from ‘I’ and even squares from triangles when only a single activated electrode differed in position (16/16 correct, figure 1*e*,*f*). Patient 2 correctly distinguished four letters individually presented randomly in 4AFC-mode (e.g. C,I,L,O (36/36), I,L,V,T (10/12)) in repetitive tests on different days (see electronic supplementary material, chapter 5, movie 2). He also distinguished sequential stimulation in clockwise versus anticlockwise direction (15 of 16 tests correct).

### (b) Light pattern perception with the microphotodiode array

The light-sensitive MPDA chip was operated at a sampling rate of 1 to 20 Hz with a pulse duration (PD) of 1–4 ms. The patient's head was comfortably positioned on a chin-rest (set up 1, figure 3*a*), and refraction was corrected for the viewing distance of 60 cm. Chip settings were adjusted for a working range of 8–800 cd m^−2^ white light or 1.2–4.3 cd m^−2^ red light (for details see electronic supplementary material, chapter 2*g*). All standardized testing was performed using a functional baseline control, i.e. performance was also tested with the chip switched off at random intervals unknown to patient and observer, as summarized in electronic supplementary material, table ST1.

#### (i) Light perception and localization

All three patients were able to perceive light mediated by the chip. This was verified in task 1, using the BaLM-test in set up 1 (figure 3*a*):

— *BaLM flash test*: in task 1, the whole screen was illuminated briefly with one or two flashes (200 ms duration with 600 ms pause) after an auditory signal. All three patients passed this test for light detection (81.3%, 100% and 100% correct, respectively) and scored well-above chance rate; (*n* = 16; ON versus OFF: *p* = 0.00005, *t*-test).
— *BaLM localization test*: when testing the ability to localize large bright areas in the visual field (small triangle in relation to a central fixation point in BaLM test) only patient 2 (87.5%; *n* = 16) passed the test successfully.
— *BaLM movement test*: perception of movement was tested with a random dot pattern at an angular speed of 1.11° s^−1^ moving in one of four directions (dot diameter 1.4 cm, average distance 1.5 cm (s.d. 0.26)), passed only by patient 2 (8 of 12, 4AFC, figure 3*e*).

In task 2, spatial resolution was tested using grid patterns (figure 3*b*). Bright lines of 0.6 cm width separated by 1.8 cm wide dark lines as well as bright lines of 0.8 cm width separated by 2.4 cm wide dark lines were presented at 63 cm distance. The orientation of these patterns was correctly recognized by patient 2. In terms of spatial frequency this corresponds to 0.46 cycles deg^−1^ (five of eight correct, 4AFC, *p* = 0.02) and 0.34 cycles deg^−1^ (four of four correct, 4AFC, *p* = 0.004), respectively (see electronic supplementary material, chapter 3, table ST1 and chapter 5, movie 3). Patient 3 succeeded at 0.22 cycles deg^−1^ (12 of 20, 4AFC, white light). Patient 1 had difficulty seeing the stripes, probably owing to her nystagmus, but distinguished horizontal from vertical lines projected onto her chip in a special set up using a fundus camera with comparable spatial arrangements and luminance.

As the spectral sensitivity of the chip is practically flat far into the infrared region, patients at several instances reported high sensitivity to infrared light.

#### (ii) Landolt C ring

In task 3, single letters and Landolt C rings were presented on the screen in various sizes (figure 3*c*). Patients 1 and 3 discerned neither the Landolt C rings nor the letters and were accordingly not presented with tests of higher difficulty in set up 1. Patient 2, the only one with the chip placed under the macula, was quite successful and his visual performance is therefore described in greater detail below.

Optimizing his implant settings resulted in an image recording time of 0.5 ms with a 7.5 Hz repetition frequency at a target luminance of 3.4 cd m^−2^ (red light), viewed with a correction of +7.0 dpt sph., −1.50 dpt cyl. at 121°. Landolt C rings (figure 3*c*) were presented in an up-and-down staircase procedure (FrACT [25]; for details see electronic supplementary material, chapter 2). A maximum of 60 s was allowed for the patient to find each C-ring on the screen in his small visual field; failure to respond in time counted as mistake. Maximum visual acuity was up to 20/1000 (log MAR =1.69)—corresponding to a Landolt ring with 4.5 cm outer diameter and a gap of 9 mm, viewed at about 60 cm distance. In three other trials on different days he achieved log MAR values of 1.75, 1.94 and 1.86, respectively (see electronic supplementary material, chapter 5, movie 4).

Patient 2 reliably differentiated also the letters L,I,T,Z on a screen (22 of 24, 4AFC, figure 3*d*; 8.5 cm high, 1.7 cm line width, corresponding to a height of approximately 9° of visual angle). He reported that having once found a letter, it appeared clearly in its natural form and was visible as a complete entity—even during its first presentation.

#### (iii) Recognition of objects on a table

In the fourth task, the ability to perceive more naturalistic scenes was tested by a standardized set up at a dining table, assessed by an independent, professional mobility trainer (figure 4*a*, for details see electronic supplementary material, chapter 2*g*). Patient 1 reliably localized a saucer, a square and a cup on the table; patient 3 correctly localized and differentiated a large plate from a saucer.

Patient 2 localized, and moreover recognized and correctly differentiated square-, triangle-, circle-, rectangular-, and diamond-shapes, which differed only in shape but not in area from each other (figure 4*b*, five of five correct, see electronic supplementary material, chapter 5, movie 5). Furthermore, he could localize and describe correctly a spoon, a knife, a cup (see electronic supplementary material, chapter 5, movie 6), as well as a banana and an apple (see electronic supplementary material, chapter 5, movie 7). Unlike the other dining table set ups this set up was entirely unknown to the patient and he was forced to make sense of an unfamiliar scene.

#### (iv) Optional tasks with letters, clock, grey papers of varying shades

The fifth group of tasks was performed only in patients who had successfully passed previous tasks. Patient 2 was able to distinguish between 16 different letters cut from white paper (5–8 cm high, font: Tahoma), placed on the black table (see figure 4*c*, 22/36 correct). The patient read letters (LOVE, MOUSE, SUOMI, etc.) correctly (five of five), also repeatedly on several days. He noted spelling mistakes in his name MIIKKA (mentioning that one ‘I’ and one ‘K’ were missing) when he first saw this word (see electronic supplementary material, chapter 5, movie 8), i.e. he perceived both individual letters and continuous, meaningful words—a prerequisite for reading.

As an additional task, a clock face was presented with two hands (6 × 1.5 cm for the hours, 12 × 1.5 cm for the minutes, figure 4*d*). Patient 2 was asked to indicate clock times set to full quarter hours. The patient correctly recognized 11 of 12 possible settings. Patient 2 also distinguished seven out of nine contrast differences of 15 per cent among nine neighbouring cards (10 × 10 cm, presented in 2AFC mode, *p* = 0.07) with linearly scaled shades of grey varying from 3 to 35 cd m^−2^ (figure 4*e*).

All patients showed distinct learning effects which, while they could not be quantified in this first pilot study, are reported as ‘spontaneous observations’ in electronic supplementary material, chapter 3*d*.

#### (v) Pupillary reflexes

Pupillary constriction in response to light as an objective measure of MPDA efficacy was assessed by infrared pupillography (for methods and recordings, see electronic supplementary material, chapter 2*i*). The amplitude of pupillary constriction was clearly more pronounced when the chip was activated (see electronic supplementary material, chapter 2, figure S2). In all three patients the chip-on condition improved pupil reaction and was always accompanied by subjective light perception. An analysis of variance was calculated for the constriction amplitudes of all three patients (with chip-on or chip-off) and patient as factors (sum of squares 0.184, *F* = 6.48, *p* = 0.022).

## 6. Discussion

### (a) The general approaches to retinal prostheses

A number of research groups have taken up the challenge of developing a retinal prosthesis. Rizzo *et al.* [4] and Weiland *et al.* [26] have reported on first trial stimulations of the retina with single epiretinal electrodes. Chow *et al*. [27] were the first to subretinally implant well-tolerated multiphotodiode arrays, intending to use the energy created by incident light for neuronal stimulation directly without amplification. However, owing to insufficient energy from the small light sensors these failed to restore vision. Second Sight (Medical Products Inc., Sylmar, CA) has a multicentre study running with the epiretinal ARGUS II device with 60 electrodes; some patients were reported to recognize large single letters by scanning them with rapid head movements [28]. Clinical studies with epiretinal electrode arrays were also performed by Koch *et al.* [29] and Richard *et al.* [30]. Other groups developed approaches with electrodes placed between sclera and choroid [8,10]. These groups argue that this ‘suprachoroidal’ approach may have the benefit of being less invasive, therefore bearing fewer risks in terms of surgical procedures. At this time, as only limited peer-reviewed information is available from ongoing clinical trials using subretinal, epiretinal and suprachoroidal approaches, it is too early to compare the final long-term outcome of the various designs. All have inherent theoretical advantages and disadvantages; basic differences and their consequences are pointed out in the following.

*Epiretinal implants* seek to interact directly with the retinal output neurons; the image processing of the complex inner retinal network must be performed externally. The processing of camera-captured images can be more easily adjusted to account for individual electrode thresholds. However, the number of simultaneously addressed electrodes is limited by present technology. Several groups have developed externally powered, fully implantable epiretinal systems with arrays of up to 60 microelectrodes [7,28–32]. Although they have reported promising results, even for long term use, the low number of electrodes limit visual performance to object localization and shape perception [33]. Yanai *et al*. [6] reported no difference in patient performance when a single pixel or multiple pixels were activated using a prototype of the ARGUS I implant. In *epiretinal* implants that use head mounted cameras, eye movements are not correlated to the visually perceived scene. Such a mismatch of visual and proprioceptive information must render object localization difficult [34].

*Subretinal approaches*, in contrast, replace in principle only the lost function of diseased photoreceptors; thus, the remaining network of the inner retina can be used for more natural processing of the image as it is forwarded, point–by-point, several times per second to inner retinal neurons. Although the surgical procedure may be more demanding, the number of pixels can be much higher, presently limited only by the size of an implant and the spatial spread of electrical stimulation. Fixation of the chip in the subretinal space is easier and, once positioned, the chip remains in place, tightly connected to the inner retina without the need for scleral tacks as used in epiretinal approaches. Moreover, our subretinal implant (Retina Implant AG, Reutlingen, Germany) is the only one so far, where the image receiver array moves exactly with the eye. This has practical implications, outlined below, as natural eye movements can be used to find and fixate a target. On the other hand, the duration of our study was limited owing to time constraints of a transdermal cable; other studies have reported longer implantation times [33]. Moreover, the range of variations in online image processing is small in devices that work quasi-autonomously under the retina.

*Suprachoroidal implants*, although bearing lower surgical risks, are located further away from target cells. This may result in high stimulation thresholds, increased power consumption, and certainly loss of spatial resolution. While the surgery is easier and less invasive, the location between highly light absorbing sclera and choroid does not allow the implantation of a light sensitive array that moves with the eye.

In the following sections, the results obtained in our subretinal study are discussed in more detail.

### (b) The spatial domain

Using simulated prosthetic vision Perez *et al*. [35] have shown that the precision in recognition tasks with normal sighted subjects increased with a density of pixels up to 1000 in a 10° × 7° visual field on the retina. Thus, at least several hundred electrodes should be employed to provide significant vision—a daunting technical barrier [35]. The present study—the first to successfully employ electronic arrays with such a large number of electrodes—presents proof-of-concept that such devices can restore useful vision in blind human subjects, even though the ultimate goal of broad clinical application will take time to develop.

The size of the visual field (11° × 11°) in our patients, although small, is sufficient for orientation and object localization, as is well established in patients with peripheral retinal dystrophies. Reading requires a field of 3 by 5 degrees according to Aulhorn [36].

Inter-individual variations in visual performance among the patients of this study can be assumed to result from their respective stages of degeneration [2], the duration of their blindness, and the retinal localization of the implant, although presently no convincing correlation can be established. Clearly spatial reorganization of the retina takes place; however, it is very slow, taking decades. As the inner retina is not dependent on choroidal perfusion, it also survives the complete loss of the choroid—as seen in our patient with choroideraemia. This also explains why blockage of choroido-retinal transport by our implant does not affect survival of the inner retina.

In our study, precise localization of the microelectrode array under the fovea appeared important for the restoration of useful percepts via spatially ordered electrical stimulation. High spatial resolution and the ability to read are restricted in normal observers to the central retina (5° × 3°), which is significantly over-represented in the visual cortex relative to more peripheral areas of the retina.

### (c) The temporal domain and the problem of image fading

Temporal resolution was investigated over a range from 1 to 20 Hz. When applying continuous electrical stimuli via the *DS-array* at a fixed retinal location with PD of 1–4 ms, patient percepts faded after approximately 15 s when presented at a 0.3 Hz repetition rate; after approx. 2 s at 2 Hz; and after approx. 0.5 s at 10 Hz. This is in close accordance with the observations of Perez *et al*. [37] with *epiretinal* ARGUS II devices that an image stabilized on the retina quickly disappears; to restore the image required a movement of the image across the retina, by means of rapid head shaking. Similarly, Jensen & Rizzo [38] observed in rabbit retina that the retinal response to a second or third electrical pulse rapidly decreases as compared to the first pulse with increasing repetition rates; apparently inner retina neurons suffer from a prolonged inhibition if stimulated electrically under conditions where the surrounding network under the electrode is being activated as a whole. By contrast, objects like grating patterns or letters can be perceived continuously with our light sensitive *subretinal MPDA*. Patients see the image constantly as a complete entity without head movements—even on the first day of stimulation. The source of this difference can be found in involuntary eye movements controlled by the superior colliculus. Even during fixation, our eyes continuously make slight movements (slow drifts and microsaccades up to 50 min of arc and 1 to 3 Hz) that refresh the image by constantly changing the activated photoreceptor population—even during strict fixation [39]. Objects viewed by our patients—with the chip moving in synchronization with natural eye movement—dynamically activate a range of adjacent pixels on the chip, as eye movements and microsaccades continuously shift the ‘electrical image’ on the retina for about 1–3 pixels, thus preventing mechanisms of local adaptation and image fading. Details on the role and magnitude of microsaccades in relation to pixel size are outlined in electronic supplementary material, chapter 3*e* (figure S3).

### (d) The cellular ‘interface’

*In vitro* experiments have shown that subretinal stimulation, at least at threshold, preferentially stimulates bipolar cells [15,19]. This may be one reason for the correct retinotopic perceptions reported in this study, since local excitation of small groups of bipolar cells is recognized in the brain at the correct position in the visual field. By contrast, epiretinal stimulation of ganglion cell fibres may result in disparities between stimulation location and perceived visual field location because the axons of RGCs course across the retina on their way into the brain via the optic nerve. On the other hand, none of the different approaches has principal problems with addressing simultaneously ON and OFF neurons (see electronic supplementary material, chapter 3*c*).

### (e) Learning and cognition

With the subretinal approach and its retinotopically correct spatial transmission, no long-term learning procedure was necessary to enable the patients to recognize shapes correctly. Even at the first trial with the DS test field or with the MPDA, patients were able to correctly perceive the complete entity of an object in the presented physical geometric form, the bright parts appearing whitish or yellowish, the dark parts as grey or black; there were no reports on colour sensations although in very rare and brief instances coloured tinges were noticed by patients.

The observation that patient 2 could readily name an object upon its first presentation to his visual field is of particular importance, and is in line with our observation of retinotopically correct perception from DS experiments and from the other patients who recognized a line and its direction clearly. This does not mean that the patients had undisturbed percepts. Patients reported some wobbling of the image, probably owing to a relatively low image capture frequency (5–7 Hz) to which they adapted quickly.

As expected, patient performance improved over time. Practising with the MPDA between 4 to 6 h daily, they had to learn to control their eye position because each object was presented within a relatively small field of vision (11° × 11°). Patient 2 reported that the two lines of the letter L were initially moving slightly independently of each other, but that they appeared connected at the corner after approximately one week. Apparently the binding of correlated motion cues can be regained quickly (see electronic supplementary material, chapter 3*d* and chapter 5, movie 9). If patients were asked to point to an object they had discovered there was clearly improvement of visuomotor abilities within a week.

### (f) Future concepts

*Methodological and technical aspects*: our first approach was designed as a short duration study of up to several weeks in only a few patients in order to achieve a proof-of-concept for a cable bound version of a subretinal active implant. Our ongoing follow-up study is employing the next-generation system (Alpha IMS; [40], produced by Retina Implant AG, Reutlingen, Germany), where an encapsulated secondary coil for power and signal transmission is positioned subdermally behind the ear, with a primary coil clipped magnetically on top. We also anticipate that lateral processing in terms of mutual inhibition of pixels, as performed in centre-surround receptive field processing will improve contrast vision and spatial resolution. Penetrating three-dimensional electrodes as developed by various groups may improve the contact to the bipolar cell layer but may be more damaging to the retina.

## 7. Conclusion

This study demonstrated that subretinal micro-electrode arrays can restore visual percepts in patients blind from hereditary retinal degenerations to such an extent that localization and recognition of objects can provide useful vision, up to reading letters. Despite all remaining biological and technical challenges, our results offer hope that restoration of vision in the blind with electronic retinal prostheses is a feasible way to help those who cannot profit from emerging gene therapy and/or the application of neuroprotective agents. The advantage of our approach is that all parts of the device can be implanted invisibly in the body, that inner retina processing can be used and that a continuous, stable image with unmatched spatial resolution is perceived. Still further development is necessary to provide long term stability, improved contrast, spatial resolution and increased field size through multiple chip implantation. Nevertheless, the present study provides proof-of-concept that electronic subretinal devices have the potential to improve visual function from a state of complete blindness to one of low vision that allows localization and recognition of objects up to reading capability.
